# Association of Opioid Use With Cardiometabolic Disease Risk Factors: Evidence From the 2009-2018 National Health and Nutrition Examination Survey

**DOI:** 10.7759/cureus.8719

**Published:** 2020-06-20

**Authors:** Kaushal Shah, Viraj V Joshi, Nkechi C Arinze, Krishna Priya Bodicherla, Shristee Ghimire, Romil Singh, Venkatesh Sreeram

**Affiliations:** 1 Psychiatry, Griffin Memorial Hospital, Norman, USA; 2 Neuropsychiatry, California Instititute of Behavioral Neurosciences and Psychology, Fairfield, USA; 3 Internal Medicine/Community Medicine, Mercer University School of Medicine, Macon, USA; 4 Psychiatry, Sri Devaraj Urs Medical College, Kolar, IND; 5 Internal Medicine, University of Science & Technology Chattogram, Chattogram, BGD; 6 Internal Medicine, Metropolitan Hospital, Jaipur, IND; 7 Psychiatry, Harlem Hospital Center, New York, USA

**Keywords:** alcohol, opioid, cocaine, methamphetamines, heroin, marijuana, illicit drugs, cardiometabolic disease, diabetes, hypertension

## Abstract

Objective

To investigate the association between opioid drug use and cardiometabolic risk factors in an adult sample data acquired from the National Health and Nutrition Examination Survey (NHANES).

Methods

A retrospective cross-sectional analysis was performed using the data from the NHANES for the period 2009-2018 provided by the Centers for Disease Control and Prevention (CDC), amounting to a total of N = 10,032 eligible participants. The data were analyzed to study the relationship between opioid drug use (dividing into four dichotomy groups: drug use (DU) group, illicit drug use (IDU) group, repeated drug use (RDU) group, and current drug use (CDU) group) and cardiometabolic disease risk factors (CDRF) (i.e., hypertension, abnormal triglyceride levels, low-level of high-density lipoproteins (HDLs), high waist circumference, insulin resistance, serum cotinine levels, higher C-reactive protein, hypercholesterolemia, and increased BMI). The statistical correlation was evaluated using the chi-square analysis, and a p-value of less than 0.05 was considered statistically significant. Alcohol use, age, race, ethnicity, education level, and poverty to income ratio (PIR) were analyzed as covariates.

Results

Overall, our analysis found that males were more likely than females (p ≤ 0.001) to have ever reported using drugs at least once in their lifetime. In fact, males were more likely than females to report ever using cocaine (p = 0.01), heroin (p = 0.01), and marijuana (p = 0.01). Additionally, males were significantly more likely than females to disclose the current use of illicit drugs (p = 0.002), and also tend to have consumed more with at least 12 alcoholic beverages per year (p < 0.001). Overall, we found no association between substance use and having a cluster of three or more CDRF variables for both males and females.

Conclusion

Study results highlight the prevalence of gender differences in DU and its reporting. With the rising popularity of illicit drugs, clinicians must be aware of its association with CDRF.

## Introduction

Cardiometabolic syndrome (CMS) is a group of metabolic dysfunctions mainly characterized by impaired glucose tolerance, insulin resistance, hypertension, dyslipidemia, and central adiposity. The CMS is a disease now recognized as an entity by the World Health Organization (WHO) [1,2]. The prevalence of illicit drug usage, cardiovascular diseases, and diabetes increased over the past decade. Heart disease is the leading cause of death in the United States (US), with an estimate of 0.64 million deaths registered each year. Per the report of the Center for Disease Control and Prevention (CDC), heart diseases cost about $219 billion each year from 2014 to 2015. Trends show a rise in the prevalence of diabetes and pre-diabetes in adults. National Diabetes Statistics estimates about 30.3 million adults diagnosed and undiagnosed diabetes in 2015, which constitutes 9.4% of the US population. Similar diabetes trends also observed amongst children and adolescents, with estimated diabetes incidence in 2015 of about 0.2 million under the age of 20 [1].

The 2016 report of National Survey of Drug Use and Health (NSDUH) estimated 44.7 million (18.3%) adults above age 18 with any mental illness (AMI), 19 million (7.8%) had substance use disorder (SUD) during the past year, and 8.2 million adults had both. Only 35 million (14.4%) adults received mental health care, and about 9.2 million adults did not receive it. Approximately 19.9 million (8.8%) adults needed treatment for substance use in the past year, out of which only 2.1 million were able to seek specialty treatment at the inpatient hospital or addiction rehabilitation facility for drugs or alcohol. About 0.66 million, 0.63 million, 0.50 million, and 0.39 million adults received treatment during the past years for marijuana, heroin, cocaine, and methamphetamine, respectively [2]. Based on the 2018 report of the Substance Abuse and Mental Health Services Administration (SAMHSA), a total of 57.8 million (26.9%) adult Americans had mental or substance use disorder or both. Among those, substance use disorder consists of 19.3 million (7.8%), and mental illness is 47.6 million (19.1%). Adults with both substance use disorder and mental illness add to 9.2 million (3.7%) of the population [3-5].

Perceived risk has dropped in the use of marijuana smoking, cocaine, and heroin in 2018 compared to 2015 [3]. Overall, the number of adults initiating marijuana, cocaine, and heroin increased in 2018. In comparison to the year 2002, the trend shows increased use in marijuana, heroin, and methamphetamine by adults above age 18 during the past year. A slight drop is observed for cocaine use during the past year for adults. Marijuana is the most used drug and showed a 15% increase in consumption in 2018 compared to its previous year. The number of adults that used marijuana, heroin, cocaine, and methamphetamines is 43.5 million (15.9%), 0.8 million (0.3%), 5.5 million (2%), and 1.9 million (0.7%), respectively [4,5]. In 2012, the reported use of marijuana was 8% of the US population, which represented a five million user increase in just five years from 2012. Diabetes patients who reported alcohol use, illicit drugs, or a combination of both have been found to have an earlier onset of type 2 diabetes mellitus compared to nonusers of alcohol or illicit drugs. According to the 2012 NSDUH data, emerging adults (aged 18 to 25 years) reported the highest current use of marijuana. Adults over 26 years of age reported marijuana use as the most frequently used drug compared to other illicit drugs. In older adults aged 50+ years, the highest increase in reported current marijuana use was among 55 to 59 year olds. The proportion of current usage has tripled in the past decade in this age group [6]. These trends depict the evidence of a growing prevalence of marijuana use and need to study long-term effects since its use is not likely to stop in the near future. Marijuana use is known to increase appetite and results in a significant increase in total calories and snack food intake high in carbohydrates. This phenomenon of an increase in calorie intake due to marijuana is well document. However, a consensus has not been reached regarding the effects on cardiometabolic risk factors [6,7]. The majority of published findings regarding cardiometabolic health-related consequences of marijuana use have been centered upon exploring associations between obesity as measured by BMI. The most common finding among the limited published data indicates a lower BMI among current marijuana users [6]. Among adolescents, studies have shown that with increasing marijuana use, they are at a greater risk of becoming obese in young adulthood than those with no marijuana users. Another concluded that the risk of obesity increases with a consistent marijuana use pattern over time [8]. In an analysis of the National Health and Nutrition Examination Survey III (NHANES III) data (1988-1994), current marijuana users aged 20 to 59 years had a lower prevalence of diabetes compared to noncurrent marijuana users. Similar to the conflicting reports of marijuana use and obesity, evidence regarding the effect of marijuana use on blood pressure is unclear. A significant increase in systolic and diastolic blood pressure has been reported; yet, other studies indicate a non-significant increase among current marijuana users compared to noncurrent users [6,8]. Moreover, some reports show increased but insignificant triglyceride levels among current marijuana users compared to nonusers [6-8].

The global incidence of the cardiometabolic risk factor (CDRF) is noted to be on the increase. The changing socio-economic conditions in the world have led to a rise in obesity and diabetes prevalence, posing the risk of CMS. According to the 1988 World Health Report, about 85% of cardiovascular diseases come from low- and middle-income countries. One study projected that in 2030 about 366 million individuals would have diabetes, with 298 million in developing nations. The changing socio-economics has decreased routine exercise, which has led to a sedentary lifestyle and unhealthy diet behavior. This phenomenon, newly labeled as "lifestyle syndrome" or "New World syndrome," is considered to be causative in the rise of cardiovascular-related morbidity and mortality in developing nations. The CMS has become a global pandemic with estimates of more than 1.1 billion adults being overweight and 312 million being obese [7].

The interrelated comorbidities of impaired fasting glucose, elevated triglycerides, low-level of high-density lipoproteins (HDLs) cholesterol, abdominal obesity, and hypertension are characteristics of metabolic syndrome (CMS) [7,8]. The prevalence of CMS amongst American adolescents accounts for about one million, which is 4% of this age group. According to the Adult Treatment Panel (ATP), obesity is not a requirement for metabolic syndrome. Individuals who have BMI even over 20-27 kg/m2 with increased abdominal visceral fat can have characteristics for metabolic syndromes, such as hyperinsulinemia, insulin resistance, and increased risk for coronary heart disease (CHD) and diabetes [8]. The prevalence of documented diabetes cases has grown tremendously, from 5.1% in 1988-1994 to 6.5% in 1999-2002. The projected approximate increase in diagnosed cases will be 12% or more by 2050 [8]. As per the data of the National Health and Nutrition Examination Survey (NHANES), the age-adjusted prevalence of metabolic syndrome increased from 24.1% in 1988-1994 to 27.0% in 1999-2000 [8]. Age-adjusted prevalence increased significantly by 23.5% (p = 0.02) in women and increased by 2.2% (p = 0.8) in men, and much of the increased prevalence, particularly in women, was due to increased prevalence of abdominal obesity assessed by waist circumference (which increased from 38.3% in 1988-1994 to 44.0% 1999-2000 in the overall population), high blood pressure (which increased from 32.2% to 39.2%), and high triglycerides (which increased from 30.2% to 32.6%). In another analysis of NHANES III data (1988-1994), metabolic syndrome prevalence was 5% and 6% in normal-weight men and women (BMI: 25 kg/m2), 22% and 28% in overweight men and women (BMI: 25 kg/m2), and 60% and 50% in obese men and women (BMI: 30 kg/m2). Ethnicity also assumed to play a role in the development of the metabolic syndrome. In NHANES III (1988-1994), Mexican Americans had the highest prevalence, 31.9%; by comparison, the prevalence in whites, African Americans, and individuals whose race or ethnicity was reported as "other" was 23.8%, 21.6%, and 20.3%, respectively. Mexican American women had a 26% higher prevalence than Mexican American men (35.6% vs. 28.3%), and African American women had a 57% higher prevalence than African American men (25.7% vs. 16.4%). Prevalence was similar in white women and white men (22.8% vs. 24.8%) [8].

Several studies have highlighted the association between illicit substance use and mental illness. The Health and Human Services (HHS) has shown the importance of addressing mental illness associated with substance use through federal funding and agencies, the National Institute of Health (NIH), CDC, and Substance Abuse and Mental Health Services (SAMHSA) [6]. Even though with these efforts, we have not noticed much progress in addressing CDRF associated with illicit drug use. CDRFs include obesity, low-density lipoproteins (LDLs), triglycerides, HDLs, blood pressure, and insulin resistance [7]. Individual studies helped us to determine several associations, such as cocaine and heart disease, cocaine and blood pressure, marijuana and stroke, marijuana and heart disease, methamphetamine and heart, and substance use and obesity [6]. However, many areas are still unexplored about the long-term effect of substance use on CDRF [6,7]. Advances are required to identify the role of nutrition in substance use and CDRF. Unfortunately, literature so far has failed us to understand this missing link [2-8]. Our objective is to close the gap by proposing a hypothesis of an association between illicit substance use and CDRFs.

## Materials and methods

Study population

The analysis was performed on the data from the NHANES. The sampling design used in this data is stratified, multistage probability cluster sampling [9]. A retrospective cross-sectional study was conducted using the data from the NHANES for the period 2009-2018 provided by the CDC. Subjects with missing data were excluded. 

To increase sample size and statistical reliability, we combined four survey periods spanning the past decade over 2009-2018 and created a dataset for analysis as recommended by the National Center for Health Statistics (NCHS) [10]. Informed consent was obtained from all patients, and the NCHS approved protocols for Health Statistics Institutional Review Board. We had a total of N = 10,032 eligible participants. Adults diagnosed with diabetes, hypertension, and pregnant females or tested positive in urine pregnancy tests were excluded from the analysis. Also excluded were self-reported subjects with the current use of hypertensive medications or insulin use.

Study variables

Potential confounding factors in between the association of opioid use and CDRF were documented including the demographics, substance use especially alcohol and tobacco use, parameters like blood pressure, cholesterol and glucose levels were quantified by using contributing factors like fasting glucose (GLU), insulin (INS), HDL, LDL, total cholesterol (TC), triglycerides (TGL), c-reactive protein (CRP), systolic blood pressure (SBP), diastolic blood pressure (DBP), and serum cotinine (Cot). Measurements of weight, height, BMI, and waist circumference (WC) were also recorded.

Cardiometabolic disease risk

Standardized cut-off values set by the International Diabetes Federation and Adult Treatment Panel III (ATP III) of the National Cholesterol Education Program were used to document CDRF. More than or equal to three abnormal CDRFs elevate the risk for cardiometabolic disease.

As per the American Heart Association (AHA) and American Diabetic Association (ADA) guidelines, current smoking status was a considerable risk factor, and serum cotinine levels, a biomarker, were used to determine current status. From the NHANES data, Cotinine is a measure of "the prevalence and extent of tobacco use" [9].

Hypertension is defined as SBP ≥ 130 mmHg or DBP ≥ 85 mmHg. Abnormal triglyceride levels were identified as > 150 mg/dl. Low HDL was identified by HDL < 50 mg/dL in men and HDL < 40 mg/dL in women. High waist circumference was defined as WC ≥ 94 cm (37 inches) for males, whereas WC ≥ 80 cm (31.5 in) for females. Insulin resistance is identified by GLU ≥ 100 mg/dL or current insulin or oral hypoglycemic use or INS ≥ 9 uU/mL. Other cut-off values included were serum cotinine levels > 3 ng/mL, CRP ≥ 0.5 mg/dL, TC ≥ 240 mg/dL and BMI ≥ 25 kg/ m² [7].

Drug use 

Substance use was quantified to determine the frequencies by using participants' self-reported drug use questionnaires identifying specific substance use like cannabis, heroin, methamphetamines, and cocaine, by distributing onto four dichotomous groups including (a) DU group, where the substances like cannabis, cocaine, methamphetamines or heroin were ever used in a lifetime; (b) IDU group, where the substances like cocaine, methamphetamines, or heroin were ever used in the lifetime; (c) RDU group, where the substances were ever used more than two to five times in a lifetime; (d) CDU group, where the substances were used in the last 30 days. Marijuana is not included in the IDU group, as compared to the DU group as marijuana was considered to be used by more than half the sample population in their lifetime. NDU is used as the reference group for analyses. 

Covariates

Alcohol use, age, race/ethnicity, education level, and poverty to income ratio (PIR) were analyzed as covariates. Alcohol use was quantified by self-reporting at least 12 drinks in the last year. Race/ethnicity was stratified to Hispanic White, Non-Hispanic Black, Other Hispanic, Mexican American and Other, as defined by NHANES. Education level was distributed as per the highest level of education for the participant: less than ninth grade, ninth to eleventh grade, high school graduate, some college or associate degree, and college graduate or higher. PIR was calculated to determine the socio-economic status by dividing the family income per poverty guidelines.

Statistical analysis

We used the 2009-2018 sample size to evaluate results with confidence and to address the gaps in available research. Statistical Analytic Software (SAS) version 9.4 (SAS Institute, Inc., Cary, NC) was used to perform the reported analyses. Appropriate subsample weights, clusters, and strata were accounted for using correct Survey design analysis methods. Subgrouping was done based on age group (20- to 45-year-old adults) and gender (within male and female subgroups). 

We used the survey frequencies to obtain descriptive demographic characteristics (N, weighted N, percent, and 95% CI) of the study sample. Mainly PROC SURVEYMEAND and PROC SURVEYREG procedures were used to obtain descriptive statistics and compare the means between the various subgroups. The statistical association was tested using the chi-square analysis, and a p-value of less than 0.05 was considered statistically significant. 

For regression modeling, the primary outcome of interest was the independent CDRF. The models were adjusted for age, gender, ethnicity, education level, PIR, and alcohol consumption. Gender-specific logistic regression models were created using domain analysis for each of the four drug use categories of interest, that is, DU, IDU, RDU, and CDU. 

The primary outcome of interest was a cluster of three or more individual risk factors (CDRF). Logistic regression modeling was performed, and the final model included age, gender, ethnicity, education level, PIR, and alcohol consumption. The model was checked for potential confounding, and odds ratios were reported with their corresponding 95% CI and p-values. The role of gender was studied to rule out any possible effect modification.

## Results

Of the entire sample, 58.14% were whites, 30.97% had an annual family income greater than $75,000, and 34.16% had some college degree. Males and females were almost in equal numbers, with 49.13% and 50.87%, respectively. Further information on the demographic distribution and characteristics of the NHANES 2009-2018 participants are shown in Table 1.

**Table 1:** Demographic characteristics (N = 10,032) N: sample size; CI: confidence interval; GED: general education development

Variables	N	Weighted N	Percent (95% CI)
Gender			
Male	4,775	53,034,348	49.13 (47.92-50.33)
Female	5,257	54,909,330	50.87 (49.67-52.07)
Total	10,032	107,943,678	
Ethnicity			
Hispanic	2,698	21,102,786	19.55 (18.78-20.37)
Non-Hispanic White	3,693	62,754,941	58.14 (57.03-59.24)
Non-Hispanic Black	2,086	13,670,352	12.66 (12.09-13.24)
Other	1,555	10,415,599	9.65 (9.07-10.23)
Education Level			
Less than 9th grade	622	4,760,213	4.41 (4.02-4.79)
9-11th grade	1,346	11,701,854	10.84 (10.18-11.50)
High school graduate/GED	2,173	22,030,349	20.41 (19.46-21.36)
Some college or associate degree	3,309	36,878,566	34.16 (33.02-35.31)
College graduate or above	2,574	32,488,988	30.10 (28.94-31.25)
Annual Family Income			
>$15,000	1,539	13,227,680	12.43 (11.72-13.13)
$15,000 - $19,999	650	5,617,588	5.28 (4.81-5.74)
$20,000 - $34,999	2,002	18,065,659	16.97 (16.13-17.81)
$35,000 - $54,999	1,687	18,596,274	17.47 (16.55-18.40)
$55,000 - $74,999	982	12,332,949	11.59 (10.77-12.40)
$20,000 and Over	228	2,224,747	2.09 (1.75-2.43)
Under $20,000	108	929,345	0.87 (0.67-1.07)
> $75,000	2,383	32,956,573	30.97 (29.78-32.15)

About half (51%) of the sample reported their lifetime or ever use of drugs. Also, 50.4%, 14.5%, and 6% reported the use of marijuana, cocaine, and methamphetamine at least once in their lifetime, respectively. The prevalence of current drug use ranged from 0.3% (heroin) to 14.8% (marijuana). Overall, males were more likely than females (57.1% vs. 45.2%; p ≤ 0.001) to have reported ever using drugs at least once in their lifetime. Specifically, they were more likely than females to report ever using cocaine (18.9% vs 10.3%; p = 0.01), heroin (2.8% vs. 1.2%; p = 0.01) and marijuana (56.1% vs. 44.9%; p = 0.01). Similarly, males were significantly more likely than females to report current use of illicit drugs (2.9% vs. 1.2%; p = 0.002) and also more likely to have consumed at least 12 alcoholic beverages a year (86.5% vs. 73.5%; p < 0.001). Statistical significance should be interpreted with a p ≤ 0.05 set at the 95% CI. Further precise details of drug and alcohol use are provided in Table 2.

**Table 2:** Drug and alcohol usage (N = 10,032) N: population size; n: sample size; *: p-value < 0.001; +: p-value = 0.01; ^: p-value = 0.002

Drug Use	Overall (N =10,032)			Male (n = 4,775)			Female (n = 5,257)		
	n	Weighted n	%	n	Weighted n	%	n	Weighted n	%
Ever used drug*	4,694	55,085,361	51	2,595	30,287,921	57.1	2,099	24,797,440	45.2
Ever used Cocaine+	1,298	15,678,865	14.5	821	10,001,245	18.9	477	5,677,620	10.3
Ever used Methamphetamines	551	6,526,003	6	351	4,208,160	7.9	200	2,317,843	4.2
Ever used Heroin+	180	2,172,576	2	122	1,490,587	2.8	58	681,989	1.2
Ever used Marijuana+	4,604	54,385,691	50.4	2,525	29,727,007	56.1	2,079	24,658,684	44.9
Ever used Illicit Drug*	1,367	16,403,152	15	860	10,417,035	20	507	5,986,117	11
Current Marijuana use*	1,483	15,954,626	14.8	920	9,829,876	19	563	6,124,751	11
Current Cocaine use*	157	1,616,567	1.5	113	1,080,804	2	44	535,763	1
Current Methamphetamine use^	54	529,509	0.5	39	403,797	0.8	15	125,713	0.2
Current Heroin use^	27	294,343	0.3	21	233,377	0.4	6	60,966	0.1
Current Illicit use^	215	2,200,867	2	154	1,522,764	2.9	61	678,101	1.2
At least 12 drinks last year*	6,651	76,862,329	80	3,653	42,037,649	86.5	2,998	34,824,679	73.5

Logistic regression analysis (adjusted for age in years, gender, race, ethnicity, education level, PIR, and alcohol use) showed no association between drug use (ever, current, illicit) and fasting insulin, fasting glucose, systolic and diastolic blood pressures, and c-reactive protein levels. Males and females both, ever use of illicit drugs were associated with a lower odds of having subnormal HDLs. Similarly, ever-use of any drug was significantly associated with a higher odd for elevated waist circumference in both genders but a lower odd for elevated BMI among females alone (OR = 0.80; 95% CI = 0.65-0.97; p = 0.024). However, ever use of illicit drugs was associated with higher odds of elevated total cholesterol for males (OR = 1.37; 95% CI = 1.03-1.82, p = 0.030) but not for females and also associated with a higher odd for elevated triglycerides among females (OR = 1.73; 95% CI = 1.07 - 2.79; p = 0.024) but not among males. Overall, we found no association between drug use and having a cluster of three or more CDRFs for both males and females, as per the analysis of CDRFs shown in Table 3.

**Table 3:** Regression analysis of each cardiometabolic disease risk factors Analysis adjusted for gender, race, ethnicity, education level, poverty to income ratio (PIR), and alcohol use. Abbreviations: OR: odds ratio; CI: confidence interval; HDL: high-density lipoproteins; LDL: low-density lipoproteins; CDRF: cardiometabolic disease risk factors

Cardiometabolic Disease Risk Factor	Drug (Ever Use)		Illicit Drug (Ever Use)		Current Illicit Drug (>2-5 times)		Drug (No Use)	
	OR (95% CI)	p	OR (95% CI)	p	OR (95% CI)	p	OR (95% CI)	p
Waist Circumference, cm								
Males	1.34 (1.06-1.68)	0.014	1.27 (0.95-1.70)	0.098	0.67 (0.11-3.87)	0.655	1.21 (0.95-1.55)	0.118
Females	1.36 (1.11-1.67)	0.003	1.33 (0.96-1.83)	0.080	0.71 (0.16-3.94)	0.677	1.25 (1.02-1.54)	0.026
Body Mass Index, kg/m2								
Males	0.86 (0.68-1.08)	0.201	1.05 (0.79-1.40)	0.717	2.02 (0.45-9.01)	0.357	0.99 (0.78-1.27)	0.979
Females	0.80 (0.65-0.97)	0.024	0.76 (0.56-1.04)	0.097	2.98 (0.72-10.56)	0.908	1.12 (0.92-1.35)	0.252
C-Reactive Protein, mg/dL								
Males	1.09 (0.74-1.60)	0.664	1.14 (0.70-1.85)	0.580	0.59 (0.01-3.58)	0.801	0.80 (0.53-1.21)	0.298
Females	1.04 (0.79-1.38)	0.768	1.47 (0.97-2.23)	0.064	0.70 (0.21-3.61)	0.953	0.95 (0.71-1.26)	0.728
Total Cholesterol, mg/dL								
Males	0.92 (0.72-1.19)	0.541	1.37 (1.03-1.82)	0.030	0.42 (0.09-1.81)	0.248	1.06 (0.82-1.37)	0.644
Females	1.01 (0.75-1.36)	0.95	0.98 (0.65-1.47)	0.938	1.12 (0.88-2.33)	0.789	1.08 (0.80-1.47)	0.58
LDL Cholesterol, mg/dL								
Males	1.08 (0.88-1.34)	0.447	1.18 (0.91-1.52)	0.201	1.83 (0.35-9.47)	0.466	1.10 (0.88-1.37)	0.392
Females	0.86 (0.70-1.04)	0.127	0.59 (0.43-0.82)	0.002	2.01 (0.90-9.85)	0.704	1.16 (0.95-1.41)	0.133
HDL Cholesterol, mg/dL								
Males	0.69 (0.58-0.82)	<0.0001	0.79 (0.64-0.98)	0.038	1.62 (0.45-5.75)	0.456	1.28 (1.06-1.54)	0.009
Females	0.79 (0.65-0.96)	0.017	0.71 (0.52-0.97)	0.037	0.89 (0.52-1.10)	0.394	1.01 (0.83-1.23)	0.9
Triglycerides, mg/dL								
Males	1.11 (0.85-1.44)	0.456	1.17 (0.85-1.60)	0.313	0.44 (0.11-1.71)	0.237	0.96 (0.72-1.26)	0.769
Females	1.15 (0.84-1.59)	0.378	1.73 (1.07-2.79)	0.024	1.15 (0.32-2.01)	0.905	0.98 (0.72-1.34)	0.923
Fasting Glucose, mg/dL								
Males	0.95 (0.76-1.19)	0.663	1.00 (0.76-1.32)	0.978	0.76 (0.15-3.77)	0.74	1.02 (0.81-1.29)	0.837
Females	0.79 (0.62-1.02)	0.074	0.92 (0.61-1.39)	0.696	0.61 (0.09-3.34)	0.691	1.11 (0.87-1.42)	0.378
Fasting Insulin, uU/mL								
Males	0.83 (0.65-1.07)	0.15	0.79 (0.59-1.05)	0.111	0.37 (0.08-1.74)	0.212	1.18 (0.92-1.53)	0.185
Females	0.86 (0.69-1.08)	0.203	0.73 (0.51-1.04)	0.087	0.22 (0.07-1.48)	0.176	1.21 (0.97-1.51)	0.088
Systolic Blood Pressure, mm Hg								
Males	0.96 (0.77-1.20)	0.735	1.00 (0.77-1.30)	0.974	2.91 (0.77-10.9)	0.115	1.08 (0.85-1.36)	0.504
Females	0.77 (0.57-1.04)	0.084	1.00 (0.63-1.58)	0.999	6.27 (0.98-9.62)	0.045	1.43 (1.05-1.93)	0.021
Diastolic Blood Pressure, mm Hg								
Males	0.91 (0.69-1.18)	0.465	0.96 (0.69-1.33)	0.819	0.41 (0.06-2.46)	0.33	0.95 (0.72-1.24)	0.718
Females	1.142 (0.809-1.613)	0.45	1.24 (0.73-2.10)	0.414	1.39 (0.18-2.03)	0.901	1.05 (0.73-1.49)	0.783
Serum Cotinine, ng/dL								
Males	3.46 (2.92-4.15)	<0.0001	3.41 (2.80-4.14)	<0.0001	1.42 (0.40-4.98)	0.581	0.23 (0.19-0.28)	<0.0001
Females	4.53 (3.79-5.42)	<0.0001	4.25 (3.29-5.50)	<0.0001	2.13 (0.45-5.38)	0.741	0.17 (0.14-0.21)	<0.0001
Cluster of ≥ 3 CDRF factors								
Males	0.98 (0.75-1.29)	0.891	0.93 (0.66-1.31)	0.706	1.56 (0.22-1.80)	0.651	0.88 (0.66-1.17)	0.4
Females	1.06 (0.84-1.35)	0.626	1.01 (0.68-1.50)	0.942	1.71(0.37-1.96)	0.75	0.88 (0.69-1.11)	0.296

## Discussion

The present study examined the prevalence and gender differences in drug use as well as the association between illicit drug use and cardiometabolic risk factors. Our results suggest that about half of the sample population (51%) have used drugs at least once in their lifetime, while current illicit use was 2%. The prevalence of current use was lower, with 14.8% and 0.3% reporting current use of marijuana and heroin, respectively.

We found significant gender differences in drug use as males were more likely than females to report current (2.9% vs. 1.2%; p = 0.002) and ever (19.6% vs. 10.9%; p < 0.001) use of illicit drugs (heroin, marijuana). Drug use ever in a lifetime was associated with elevated WC (male: OR = 1.34, CI = 1.06-1.68, p = 0.014; female: OR = 1.36, CI = 1.11-1.67, p = 0.003) with HDL (male: OR = 0.69, CI = 0.58-0.82, p < 0.0001; female: OR = 0.79, CI = 0.65-0.96, p = 0.017) for both genders. Finally, our analysis showed no significant association (p > 0.05) between drug use and the cluster of three or more CDRF.

Our findings support the published literature that the prevalence of drug use continues to rise in the United States [11]. In our data, 14.8% reported current use of marijuana which is relatively higher than the 9.5% reported by a National Institute on Alcohol Abuse and Alcoholism (NIAAA) study 2013 [11]. Undoubtedly, cannabis remains the most widely used drug in the United States, and its use is on the rise [12]. A possible explanation for this rise is the recent legalization of medical and recreational marijuana use across the United States. Recent studies have associated the legal policy changes with increases in marijuana access and use among adults [13,14]. Another plausible reason is the rapidly expanding electronic cigarette industry, which may have altered perceptions of drug use and improved access since e-cigarettes may be used as a delivery system [15].

Our analysis also found significant gender differences in drug use. From our results, males were more likely than females to have reported drug use at least once in their lifetime. Also, they are more likely than females to be current illicit users of marijuana, cocaine, methamphetamine, and heroin. Gender differences in drug and alcohol use are well established and documented in the literature [16-20]. One hypothesis to explain this finding stems from studies that suggest that females were more likely to perceive a significantly higher risk associated with drug and alcohol use [16,21,22]. Another possible explanation originates from the published research, which shows that females have a lesser propensity for risk-taking than males [23,24]. However, these factors alone may not explain these gender differences, and more longitudinal studies are needed further to evaluate the association between gender and drug use critically.

Further, we examined gender differences in the association between drug use and cardiometabolic risk and observed interesting results. Overall, drug use (any category) was not associated with a cluster of three or more CDRF in both males and females. A history of ever use of any drug was associated with higher odds for elevated waist circumference levels and lower odds of having low HDL for both genders but associated with lower odds for elevated BMI among females only. Interestingly, the current use of illicit drugs was not associated with any cardiometabolic parameter in both genders. Substance use is considered as a risk factor for metabolic syndrome, but empirical evidence is still lacking [25]. Several studies have examined the association between drug use and cardiometabolic risk and reported protective benefits of drug use. Christin et al. found lower fasting insulin, insulin resistance, body mass index, and waist circumference in users compared with nonusers [26]. Contrary to a cross-sectional study of 8,478 US adults by Vidot et al., we did not observe a significant difference in the CDRF cluster of three or more in female drug users compared to nonusers. Our study observations are not exactly the same compared, yet it highlights the crucial issue related to drug use [27]. In another study, increased usage of marijuana use was shown to increase the odds of metabolic syndrome [28]. Most of these studies focused solely on marijuana use, and so we cannot make direct comparisons. We found some evidence of gender differences regarding drug use and body mass index, waist circumference, and cholesterol levels. The reasons for these differences are uncertain, and prior research reported conflicting findings [29,30]. More studies to elucidate the mechanisms for these differences are needed.

Although data from 2009 to 2018 were pooled to increase the analytic sample size, our study had few limitations. The NHANES is fundamentally a cross-sectional study and, as such, a longitudinal assessment of the causal relationship between drug use and cardiometabolic risk is not possible. It is also possible that self-reporting may have resulted in decreased drug use reporting, resulting in a systematic bias. Future studies should closely examine the impact of other factors such as chemical exposure, sugar intake, tobacco consumption, etc., on the association between DU on CDRF in adults.

## Conclusions

Our study provides an insight into the prevalence of illicit drug use and CDRFs. In contrast to past evidence, this study clarifies that illegal drugs are not associated with three or more CDRF. However, lifetime drug usage status shows an association with a subnormal low-level HDLs cholesterol for males and waist circumference for both genders. This study also unravels the gender report difference in the use of illicit drugs and alcohol. However, due to the cross-sectional study type, it cannot establish a causal relationship. Due to the changing pattern of drug use, eating habits, and lifestyle from the past decade, it is crucial to evaluate these findings through a prospective study. Above all, these findings are essential for clinicians to be aware of to serve their community optimally by creating awareness, taking preventative measures, and manage it to lower down the associated risks.
